# Literary Note

**Published:** 1899-12

**Authors:** 


### LITERARY NOTE.

THE current issue of *Therapeutic Notes* contains an interesting article on the propagation of vaccine, which is of special interest to the medical profession. In view of the active campaign the anti-vaccinationists are waging, it is important that physicians shall familiarise themselves with the methods employed in the propagation of animal vaccine. Most of all, they must know where to get it in a pure, uncontaminated state.

The article referred to contains illustrations in half-tone of the biological department of Messrs. Parke, Davis & Co., Detroit, showing the details of the method employed. It impresses one with the perfection of the system and gives confidence in the vaccine that results from it. The article will be furnished on application to the aforementioned house.

